# BBC3 (PUMA) regulates developmental apoptosis but not axonal injury induced death in the retina

**DOI:** 10.1186/1750-1326-6-50

**Published:** 2011-07-15

**Authors:** Jeffrey M Harder, Richard T Libby

**Affiliations:** 1 Department of Pathology and Laboratory Medicine, University of Rochester Medical Center, Rochester, NY, USA; 2 Flaum Eye Institute, University of Rochester Medical Center, Rochester, NY, USA; 3 Center for Visual Sciences, University of Rochester Medical Center, Rochester, NY, USA; 4 Department of Biomedical Genetics, University of Rochester Medical Center, Rochester, NY, USA

## Abstract

**Background:**

Naturally occurring apoptosis is a developmental process that shapes the retina by eliminating overproduced neurons. In the absence of the proapoptotic Bcl-2 family member BAX, developmental apoptosis in the retina is disrupted and extra neurons survive. It is unknown how BAX is activated or if this regulation varies between neuronal types and subtypes. Since the Bcl-2 family members BIM, BID, and BBC3 (PUMA) are powerful direct activators of BAX, we used mice deficient for each of these genes to investigate their importance in developmental apoptosis.

**Results:**

*Bax *deficient mice have an increase in retinal ganglion cells (RGCs), bipolar cells and dopaminergic amacrine cells, but not photoreceptors, horizontal cells or cholinergic amacrine cells. The retinas of adult *Bim *and *Bid *deficient mice appeared to have no increase in any retinal cell type. *Bbc3 *deficient mice, either homozygous or heterozygous for a null allele of *Bbc3*, had an increase in the same cell types as *Bax *deficient mice. An analogous result may occur in the brain where, similar to *Bax *deficient mice, *Bbc3 *deficient mice have a larger gross brain weight compared to wild type mice. In contrast to its developmental role, BBC3 did not appear to be a primary factor in BAX-dependent axonal injury induced neurodegeneration in adult RGCs.

**Conclusion:**

The regulation of BAX activation in the retina appears to be complex, dependent on the developmental stage of the animal, the nature of the insult and even the type of neuron.

## Introduction

Apoptosis in the retina has a major role in neuronal development and neurodegeneration. Retinal development is a complex process involving the cell fate commitment and differentiation of seven cell types and numerous cell-subtypes. Cell death is an important component of determining the final composition of the retina as most cell types are overproduced during development [1,2]. Numerous mechanisms have been suggested to play a role in determining the ultimate number of retinal neurons [3,4]. For instance, competition for neurotrophic support was suggested to be important in determining retinal ganglion cell (RGC) number. However, even though extensive manipulation of the neurotrophic deprivation pathway affected the rate of cell death, it did not alter the final number of RGCs [4-6]. To date it is unclear what molecular pathways are critical for determining final cell number in the retina or other parts of the central nervous system (CNS).

It is known that BAX is an important proapoptotic molecule in retinal development, after RGC axonal injury, and in glaucoma [1,7,8]. *Bax *is required in the developmental cell death of retinal ganglion cells (RGCs), photoreceptor cells, and at least some retinal interneurons [1,9]. BAX is a member of the Bcl-2 family of apoptotic regulators, and BAX activation is a final step in triggering apoptosis. Numerous other Bcl-2 family members carry out either prosurvival or prodeath functions by regulating BAX activity. This regulation carries significance in the retina where BAX overexpression alone did not alter RGC number [10]. Cell signaling pathways alter individual Bcl-2 family member activity, which ultimately determine whether BAX (or BAK1, another prodeath Bcl-2 family member that is capable of triggering apoptosis) is activated. During retinal development, it is unclear what Bcl-2 family members regulate BAX activation. To understand cell death during retinal development, it is important to define the critical steps to activating BAX.

There are at least 7 BH3-only proteins, prodeath Bcl-2 family members. Three of these, BIM, BBC3 and BID are powerful direct activators of BAX[11]. Doonan and colleagues [12] showed that BIM did not play a significant role in determining the final number of retinal neurons, though it might have a minor role in determining the rate of RGC death during development. BID is expressed in the developing retina [13], but it is not known whether it is activated (truncated) or has any role in regulating developmental cell death. BBC3 (also known as PUMA) is expressed in the developing retina [13]. Interestingly, BBC3 is known to be critical in some cells after insults that have been implicated in developmental neuronal death. For instance, BBC3 is known to play a role in certain cell types after cytokine withdrawal [14,15] and following suppression of neuronal activity [16]. Recently, BBC3 has also been shown to be critical for the death of newly generated neurons in the adult brain [17,18]. In addition BBC3 is a powerful mediator of neuronal apoptosis after various insults in the adult [19-21].

Here we examine the role of the direct activators of BAX that have not been assessed, BID and BBC3, to determine if they are important for BAX activation in retinal development. There were no obvious changes in retinal cell number in adult *Bid *deficient mice. *Bbc3 *deficiency significantly increases several different retinal cell types, including RGCs. Interestingly, RGC developmental death and death after axonal injury is BAX dependent [1,7,8,22], but *Bbc3 *deficiency only protects RGCs from developmental death. These data suggest that BAX dependent apoptotic signaling in RGCs is differentially regulated even at the level of BAX activation. Furthermore, these data define a role for BBC3 in neuronal development.

## Results

### *Bid *deficiency does not alter retinal cell numbers

**Figure 1:**
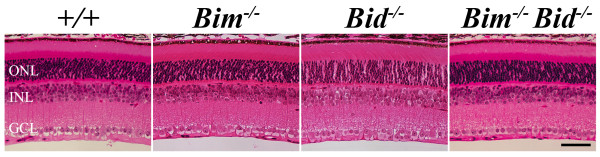
**Deficiency in *Bim *or *Bid *do not alter adult retinal morphology**. Retinal structure for both *Bim *and *Bid *deficient animals (aged 6 weeks or more) appeared to be grossly normal with no obvious increase in neuronal number in any retinal layer. Furthermore, no extra cells were obvious in adult mice that were deficient in both *Bim *and *Bid*, *Bim*^-/- ^*Bid*^-/-^. At least 3 retinas for each genotype were assessed. ONL, outer nuclear layer; INL, inner nuclear layer; GCL, ganglion cell layer; scale bar, 50 μm.

Neurons in the adult retina reside in three layers of cell bodies: the ganglion cell layer (GCL), inner nuclear layer (INL), and outer nuclear layer (ONL), which are connected by two synaptic layers: the inner plexiform layer (IPL) and the outer plexiform layer (OPL). Developmental death plays a role in the organization and final number of neurons in each layer. Both BIM and BID expression have previously been shown in the developing retina throughout the time when cell number is being refined [13]. *Bim *deficiency delayed developmental cell death throughout the retina, but did not appear to affect final cell number [12]. Retinal cross sections of adult *Bim^-/- ^*mice were normal (Figure 1). Similarly, deficiency in *Bid *did not appear to alter the number of surviving cells in the adult retina (Figure 1). Counts of total neurons in the ganglion cell layer (GCL; the inner layer of the retina) of Nissl stained retinal flat mounts confirmed that neither BIM nor BID affects cell number in the GCL (given as % wild type, WT 100 ± 8%; *Bid^-/-^*, 102 ± 4%; *Bim^-/-^*, 106 ± 4%; n = 4 for each genotype, P > 0.5). In some instances, these BH3-only proteins may act in consort with other BH3-only proteins [11,23]. Therefore we investigated whether this was true with BIM and BID. The *Bim^-/- ^Bid^-/- ^*double mutant retinas had normal morphology (Figure 1) and did not have extra retinal neurons in the GCL as judged by cell counts of retinal flat mounts (105 ± 6% of, n = 4; P > 0.5).

### *Bbc3 *deficiency increases neuronal number in the retina

**Figure 2:**
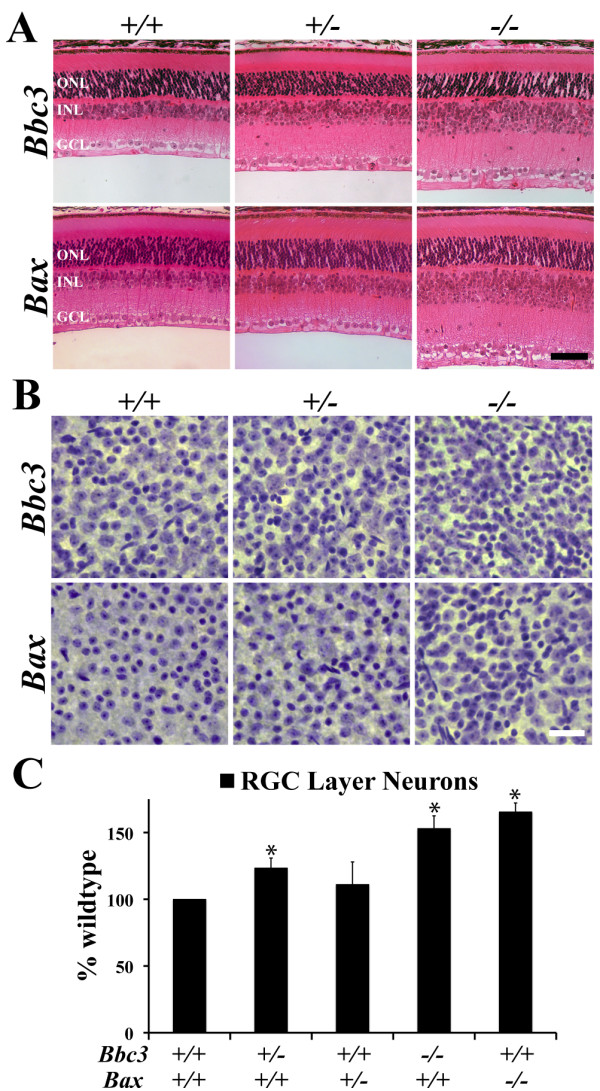
**Deficiency in *Bbc3 *increases neuron number in the adult retina**. (**A**) Histological analysis of the retinas of *Bbc3^-/- ^*and *Bbc3^+/- ^*mice (aged 6 weeks or more) reveal an increase in thickness of inner nuclear layer (INL), suggesting an increase in INL neurons. Similarly, there appears to be an increase in ganglion cell layer (GCL) neurons. The retinas of *Bax^-/- ^*mice also appeared to have an increase in INL and GCL cells. However, no obvious increase in cell number was preset in *Bax *heterozygous mice. No increase in the number of photoreceptors, which make up the outer nuclear layer (ONL) was observed in either *Bbc3 *or *Bax *deficient retinas. **(B) **To confirm the observation of an increase in GCL neurons in *Bbc3 *and *Bax *deficient retinas, GCL neurons were stained in flat mount using a modified Nissl stain. (**C**) Cell counts confirmed an increase in GCL neurons in *Bbc3*^-/-^, *Bbc3*^+/-^, and *Bax^-/- ^*neurons. At least 5 retinas were counted per genotype. Note, for cell counts, endothelial cells were excluded based on their elongated morphology. *, P < 0.05; scale bar, 100 μm in A, 50 μm in C.

BBC3 expression has been reported during the postnatal wave of developmental death in the retina [13]; therefore, we examined the *Bbc3 *null retina to see whether its presence is required for normal retinal development. Compared to the wild type retina, cross-section thickness progressively increases in *Bbc3 *heterozygote and *Bbc3 *null retinas corresponding to the loss of one or both alleles of *Bbc3 *(Figure 2A). This increase in size is due to increased cellularity in the INL and GCL and a thicker IPL (the synaptic layer between the GCL and INL) in the *Bbc3 *mutant retinas. In contrast, the ONL, which contains photoreceptor cell bodies, appears unaffected. The architecture of the *Bbc3 *null retina is consistent with the phenotype observed in the *Bax *null retina and a disruption of developmental apoptosis (Figure 2A). However, the phenotype in the *Bbc3 *heterozygote was unexpected, as there does not appear to be an increase in retinal neurons in *Bax *heterozygote mice.

In order to assess the extent of protection in the GCL, where *Bax *is known to be important in mediating developmental cell death [1,7], a modified Nissl stain was performed on retinal flatmounts (Figure 2B). The total number of GCL neurons was significantly increased in the *Bbc3^+/- ^*and *Bbc3^-/- ^*mice (Figure 2C, P < 0.01). Quantification of the *Bax^+/- ^*retinas yielded no significant difference, while in *Bax^-/- ^*retinas GCL neurons were significantly increased (Figure 2C; P < 0.01). These results are consistent with the retinal cross-sections and also indicate no significant difference between the *Bbc3 *and *Bax *knockouts in terms of the number of GCL neurons.

**Figure 3:**
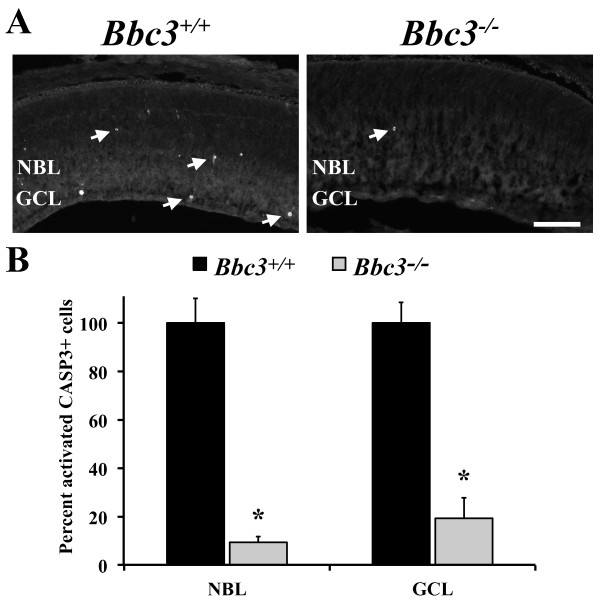
**BBC3 is important for cell death in postnatal retinal neurons**. (**A**) At postnatal day 4, *Bbc3^-/- ^*retinas have far fewer cleaved CASP3+ (dying) cells compared to wild type retinas (arrows indicate examples of activated CASP3+ cells). (**B**) The number of CASP3+ cells was significantly different in both the retinal ganglion cell layer and the developing outer retina (P = 0.001; n = 3 for each genotype). Scale bar, 100 μm.

BAX contributes to final neuronal number due to its role in postnatal death [1,24]. To confirm that BBC3 plays a similar role in postnatal cell death, dying cells were counted in postnatal day 4 retinas, a time when cells are dying in both the GCL and the developing outer retina (NBL, which consists of neuronal precursors and differentiated neuronal cells) [2,24]. CASP3 activation (cleaved caspase) was significantly reduced in both layers of *Bbc3^-/- ^*retinas compared to wild type (Figure 3; P < 0.001). Thus, BBC3 plays a critical role in the apoptotic death of retinal neurons in the postnatal retina. It is important to note that there were still occasional dying cells in both layers, suggesting another caspase-dependent cell death pathway eliminates a small number of retinal cells in the absence of BBC3.

### Inner retinal cell types that require BAX for developmental apoptosis share a BBC3-dependent cell death pathway

**Figure 4:**
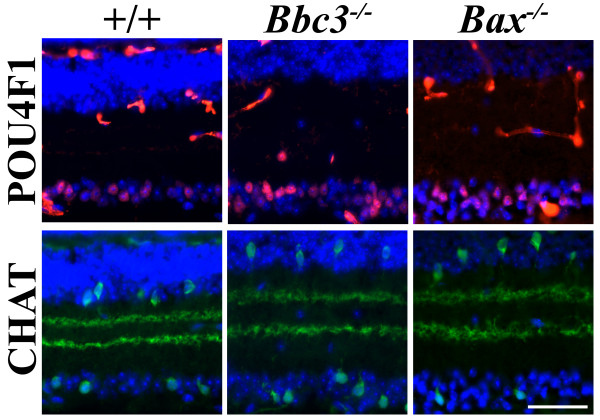
***Bbc3 *increases the number of ganglion cell layer neurons in the adult retina**. There is an increase in POU4F1 retinal ganglion cells (red) in both *Bbc3 *and *Bax *deficient retinas. The number of cholinergic amacrine cells (green) is not increased in either *Bbc3 *of *Bax *deficient retinas. Note, CHAT+ cell numbers where similar to controls in both the inner nuclear layer (INL) and in the GCL (displaced amacrine cells). Quantification of CHAT and POU4F1 positive cells are given in table 1. Scale bar, 50 μm.

The different types of retinal neurons are overproduced and eliminated in different amounts [25]. However, whether similar or different, the cell signaling pathways responsible for developmental death have not been identified in any retinal cell type. We sought to determine if differences between the *Bax *null and *Bbc3 *null phenotype could identify key cell-type specific differences in the regulation of apoptotic proteins by assessing whether all types or a subset of retinal neurons are affected in each mutant. We quantified which cell types increased as a result of *Bbc3 *or *Bax *deficiency in the GCL and INL. The ganglion cell layer contains two types of neurons, RGCs and displaced amacrine cells. POU4F1 is a specific marker for RGCs and is expressed in 80% of adult RGCs [26]. *Bbc3^+/- ^*and *Bbc3^-/- ^*mice had significantly more RGCs than wild type mice (Figure 4 andTable 1). The increase was similar to that seen in *Bax^+/- ^*and *Bax^-/- ^*mice. Both *Bbc3*^-/- ^and *Bax*^-/- ^had approximately twice the number of POU4F1+ cells. In addition, the percent of RGCs in the GCL in the mutant mice does not appreciably differ from controls (e.g., % DAPI+ GCL cells, *Bbc3*^+/+ ^36%; *Bbc3*^-/- ^41%). This result indicates that the naturally occurring cell death of displaced amacrine cells is also disrupted in the *Bbc3 *mutants. However, all displaced amacrine cells do not appear to undergo BBC3 or BAX-dependent cell death. Deficiency in either *Bbc3 *or *Bax *did not effect the final number of cholinergic displaced amacrine cells (CHAT+; Figure 4 andTable 1), which make up approximately 20% of displaced amacrine cells in the GCL in wild type mice [27].

**Table 1:** Specific Cell Counts.

		***Bbc3***		***Bax***	
	**+/+**	**+/-**	**-/-**	**+/-**	**-/-**
	
POU4F1	100 ± 5.31	**136.5 ± 2.3**	**192.5 ± 4.8**	**131 ± 3.5**	**203.4 ± 3.5**
GCL-CHAT	100 ± 2.33	93.0 ± 5.2	113.2 ± 12.8	105.8 ± 6.6	96.5 ± 7.5
GCL-DAPI	100 ± 7.8	120.9 ± 3.9	**164.2 ± 4.8**	112.2 ± 9	**190 ± 2.7**
INL-CHAT	100 ± 1.0	82.5 ± 5.4	99 ± 7.4	101.7 ± 7.4	91.4 ± 4
TH	100 ± 5.8	**194.4 ± 4.4**	**337.1 ± 3.3**	90.3 ± 1.8	**380.8 ± 7.28**
VSX2	100 ± 6.0	**121.7 ± 4.5**	**146.7 ± 3.6**	98 ± 1.9	**147.1 ± 3**
CALB1	100 ± 3.8	97.7 ± 5.	99.6 ± 1.8	93.9 ± 2.9	92.1 ± 0.7

GCL, ganglion cell layer; INL, inner nuclear layer; n, 3-5 for each genotype and cell type; Bold, P < 0.05

**Figure 5:**
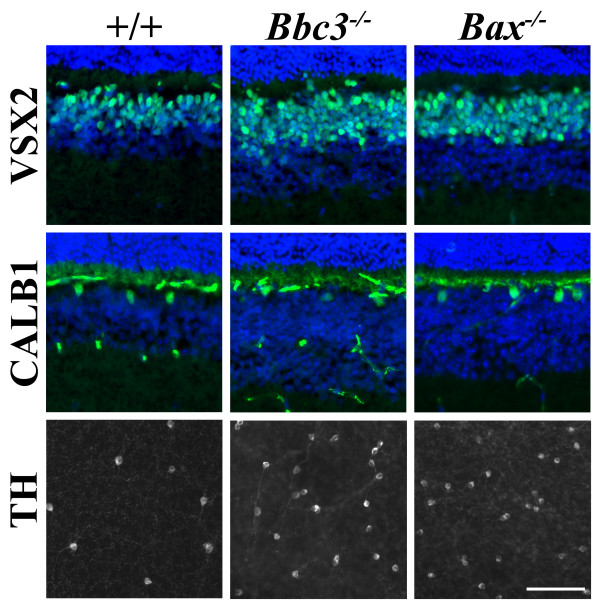
**BBC3 is important for the death of several retinal interneurons**. *Bbc3 *and *Bax *deficiency increased the number of bipolar cells (VSX2+ cells) but not the number of horizontal cells (CALB1+ cells) in the inner nuclear layer (INL). The number of dopaminergic amacrine cells (TH), which reside in the INL were assessed in flat mount since there are so few in the retina. Deficiency in either *Bbc3 *or *Bax *greatly increased the number of these cells surviving in the adult retina. Quantification of VSX2, CALB1 and TH positive cells are given in table 1. Scale bar, 50 μm.

There are three broad types of retinal neuron in the inner nuclear layer: amacrine cells, bipolar cells, and horizontal cells. As in the GCL, developmental cell death of cholinergic (CHAT+) amacrine cells in the INL did not appear to require BBC3 or BAX (Figure 5 andTable 1). However, the number of dopaminergic (TH+) amacrine cells was increased in *Bbc3*^-/- ^and *Bax*^-/- ^retinas by over three fold. Interestingly, there was a significant increase in dopaminergic amacrine cells in *Bbc3*^+/- ^but not *Bax*^+/- ^mice. Similarly, there was a significant increase in bipolar cells (VSX2+) in *Bbc3*^+/-^, *Bbc3*^-/- ^and *Bax*^-/- ^but not *Bax^+/- ^*mice. Horizontal cells (CALB1+) are not known to undergo significant amount of Bcl-2 family dependent death during development [25]. As expected horizontal cell numbers were similar in retinas of all genotypes. Overall, these results confirm the varying amounts to which different types of retinal neurons are overproduced and eliminated. Despite these differences BBC3 appears to be the primary activator of BAX in the developing retina, with the complete loss of BBC3 or BAX resulting in similar phenotypes.

### BBC3 contributes to developmental apoptosis in other regions of the CNS

Developmental apoptosis is an essential component in the proper development of the entire CNS [22]. Similar to the retina, a large amount of this naturally occurring cell death coincides with synapse formation [28]. The importance of BAX-dependent cell death pathways in the developing brain has been observed regionally and by increased gross brain weight in *Bax^-/- ^*mice compared to wild type mice and varies with sex [29]. Similarly, in *Bbc3^-/- ^*mice there was approximately a 15% increase in gross brain weight compared to wild type mice in both males and females (Table 2; P < 0.005 for each sex).

**Table 2:** BBC3 is important for developmental cell death in the brain.

		**Brain weight (mg)**	**Body weight (g)**
		
*Males*	*Bbc3^+/+^*	448 ± 5	20.7 ± 0.5
	*Bbc3^-/-^*	512 ± 16*	18.8 ± 0.4
*Females*	*Bbc3^+/+ ^*	433 ± 15	17.0 ± 0.8
	*Bbc3^-/-^*	497 ± 11*	17.0 ± 0.9

All mice used were between 52 and 64 days old. *, P < 0.005 compared to *Bbc3^+/+ ^*of same sex and the effect remains after adjusting for body weight (P < 0.001). N>5 for each sex and genotype.

### *Bbc3 *deficiency does not prevent RGC death after axonal insult

**Figure 6:**
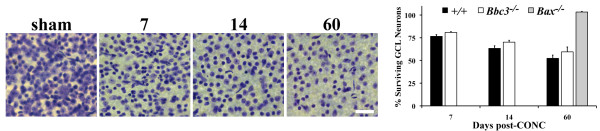
**BBC3 is not required for the death of adult retinal ganglion cells (RGCs) after axonal injury**. Nissl stained flat mounts of ganglion cell layer (GCL) neurons (left) in *Bbc3 *deficient mice shows that RGCs are still lost at 7, 14 or 60 days after CONC. Note, only RGCs die after CONC. Approximately 55% of RGC layer neurons are amacrine cells [27], so a loss of 45% of RGC layer neurons equals complete RGC loss. In contrast to *Bbc3 *deficiency, *Bax *deficiency completely protects RGCs from apoptosis after CONC even out to 60 days. N > 5 for each genotype and time point.

*Bbc3 *appears to be as critical as *Bax *in the developmental death of RGCs. Since *Bax *is required for RGC death in glaucoma [8] and after optic nerve injury (Figure 6 and [7]), *Bbc3*'s role in the death of adult RGCs from axonal insult was tested. Counts of total GCL neurons showed that significant loss of RGCs occurred in *Bbc3*^-/- ^mice at 7 and 14 days after CONC compared to the mice undergoing a sham procedure (Figure 6). This result differs from *Bax^-/- ^*mice, where no significant loss of RGCs was reported 14 days [7] and 21 days [8] after CONC. In order to assess whether the extent of RGC loss is diminished following axonal injury in *Bbc3^-/- ^*mice, RGC layer neurons were counted 60 days post CONC, which is well after the normal period of cell death. 60 days after axonal injury, wild type retinas lost 44% of RGC layer neurons (Figure 6). Note, approximately 55% of RGC layer neurons are displaced amacrine cells [27] and only RGCs die after direct axonal injury [30], so the observed loss of 44% of RGC layer neurons equals complete RGC loss. *Bax *deficiency continued to provide complete protection at 60 days, but the percentage of RGC loss that occurred with *Bbc3 *deficiency was similar to wild type (Figure 6). Unlike BAX, BBC3 only has a role in developmental death and is not a factor in long-term RGC survival after an acute axonal injury.

Counting total number of GCL neurons is a good way to determine if there is long-term protection, but the method may not be sensitive enough to detect a delay in RGC death. To determine if *Bbc3 *deficiency delayed RGC death, the number of CASP3+ cells in *Bbc3^-/- ^*and control retinas were counted at 3 (just as RGCs begin to die) and 5 days after CONC. At 3 days after CONC, the first day that significant numbers of dying RGCs are observed, there were approximately an equal number of CASP3+ cells in *Bbc3^+/+ ^*and *Bbc3^-/- ^*retinas (*Bbc3^+/+ ^*100 ± 8.1; *Bbc3^-/- ^*89.1 ± 13.5; P = 0.5; N = 9 for each genotype). Given that there are more RGCs in *Bbc3^-/- ^*retinas it is possible that BBC3 has a small role in RGC death after CONC; a caveat is that the nerve is bigger which may provide some cushion from the mechanical trauma of the injury. By 5 days there were significantly more in the *Bbc3^-/- ^*retinas compared to control, but this increase appears to be in proportion to the increase in the RGCs in these retinas (given as percent control ± SEM; *Bbc3^+/+^*100 ± 7.6; *Bbc3^-/- ^*140.5 ± 12.3; P = 0.023; N = 5 for each genotype). Thus, BBC3 does not appear to have a major role in RGC death after axonal injury.

## Discussion

In the central nervous system Bcl-2 family members are involved in developmental cell death as well as in most cell death that is the result of trauma and disease [31]. BAX is a powerful prodeath member of the Bcl-2 family. In fact BAX activation is often a critical step in triggering apoptosis. BAX activation is tightly controlled by the interaction of other Bcl-2 family members. Prosurvival family members antagonize BAX activation while BH3 only proteins, another type of Bcl-2 family prodeath proteins, facilitate BAX activation [32]. In the retina, BAX has been reported to be critical for the developmental cell death of many retinal cell types [1,9]. Furthermore, BAX activation is an important step for the death of adult retina neurons after an insult, including for axonally injured RGCs [7,8]. Currently, the complete set of Bcl-2 prosurvival and prodeath proteins that control BAX activation in developing retinal cells or in axonally injured RGCs is unknown.

Most retinal neurons are overproduced during development and BAX is involved in the programmed cell death that prunes the extra neurons [22]. The gross morphology of *Bax^-/- ^*retinas has been described [1,24]. In *Bax^-/- ^*mice there are approximately twice as many RGCs compared to wild type retinas [1,22]. Also, the inner nuclear layer of *Bax^-/- ^*retinas is much thicker than controls suggesting that there are more retinal interneurons (bipolar, amacrine and/or horizontal cells). We confirmed the involvement of BAX in RGC death. Furthermore, cell counts showed that BAX is required for the death of bipolar cells but not horizontal, which may not be overproduced in large numbers [2,25]. Interestingly, subtypes of amacrine cells differed in their requirement for BAX. BAX appears to be required for the death of dopaminergic but not cholinergic cells even though cholinergic cells are overproduced and die during postnatal development [25]. Overexpression of Bcl-2, a powerful prosurvival member of the Bcl-2 family that can antagonize all of the prodeath BH3-only proteins results in a similar phenotype as *Bax *deficiency [25] with the exception that *Bcl2 *overexpression produced far more dopaminergic amacrine cells than Bax deficiency. Thus, the Bcl-2 family appears to be important in developmental cell death of numerous retinal cell types and the importance of specific family members can vary even amongst the subtypes of a specific neuron.

BAX activation likely requires BH3-only proteins. Mice lacking the BH3-only protein BIM had delayed cell death in all retinal layers during development [12]. However, adult retinas in *Bim^-/- ^*mice appeared to have normal numbers of retinal cells [12] (and see Figure 1). Doonan et al., [12] hypothesize that other BH3-only proteins ultimately are recruited to kill developing retinal neurons. This study left unresolved whether in the normal retina a BIM-dependent pathway was responsible for killing developing retinal neurons. To determine if other BH3-only proteins had a role in retinal developmental death we focused on BID and BBC3, which like BIM antagonize all the prosurvival family members [33] and are capable of directly activating BAX, BID and BBC3 [11]. *Bid *deficiency did not affect the final number of retinal neurons. Surprisingly, since it has not been implicated in neuronal developmental cell death, BBC3 was required for death in all of retinal cell types that have BAX dependent developmental death. It appears that BAX activation during development is entirely BBC3 dependent. Thus, regulation of BBC3 appears to be a dominant factor determining a cell's commitment to die in the developing retina and the brain.

It is possible that the levels of prosurvival Bcl-2 family members relative to BBC3 (and other prodeath Bcl-2 family members) are key to determining which neurons survive naturally occurring developmental cell death in the postnatal retina. In fact, this model has been suggested to be important in RGC death after axon injury [34]. In developing retinal neurons BCL2 appears to be ubiquitously expressed [35]. BCL2 is required for the survival of a subset of RGCs after the naturally occurring window of developmental death [36], supporting the idea that neuronal survival is normally achieved by tipping the balance in favor of the prosurvival family members. However, the absence of BCL2 does not increase RGC death during the major wave of developmental cell death [36]. This result may be due to a high level of other prosurvival Bcl-2 family members in RGCs. BCL2L1 (BCL-XL) is expressed in the developing central nervous system and is required for survival of immature neurons [37,38]. Assessing the function of BCL2L1 and other prosurvival family members in the developing retina would critically test this possibility. In addition, it is notable that despite the threshold set by prosurvival family members and the presence and importance of BIM (retinal developmental death is delayed in its absence [12]), BBC3 is specifically required for death to occur. Therefore, further study of the regulation and/or specificity of BBC3 is needed to understand the processes governing naturally occurring cell death in the retina.

It is unclear how BBC3 expression is regulated in the developing retina. The effect that *Bbc3 *heterozygosity has in increasing neuronal number indicates that its transcriptional control is important. *Bbc3 *expression is known to be rapidly induced in response to various insults, including neurotrophic deprivation, by multiple transcription factors [39]. The death of newly generated neurons in the adult brain is similar to the death of developing retinal neurons in that it requires BBC3 [18,21]. However, *Bbc3 *expression in the newly generated adult neurons is dependent on a major regulator of *Bbc3 *expression, TRP53 [18]. Surprisingly, TRP53 is not the major regulator of *Bbc3 *expression because retinal cell death in development appears to be *Trp53 *independent [40]. *Bbc3 *expression is also known to be regulated by other transcription factors, including, TRP73, E2Fs, FOXO3A, JUN and MYC [39]. Knockout of many of these transcription factors *E2fs*, *Foxo3a *(unpublished data), and *Jun *(unpublished data) has no lasting effect on retinal development, at least in terms of the gross number of retinal neurons. These observations limit the number of known *Bbc3 *regulatory pathways that could be important in determining adult neuronal number.

Many physiological processes influence cell death in the developing retina and are potentially upstream of BBC3, including neurotrophin signaling, electrical activity, and refinement of spatial patterning [3,4,41]. However, manipulations of these processes differ from *Bbc3 *deficiency in that they only affect the rate of cell death and do not alter adult neuronal number (e.g. [3-6]). Interestingly, electrical activity has been suggested to be important in maintaining RGC survival in development [42] and synaptic activity can suppress *Bbc3 *expression, in a *Trp53 *independent manner, in developing neurons [16]. Though, it is unclear how electrical activity regulates *Bbc3 *activity, perhaps understanding this link may lead to the identification of molecules important in determining neuronal number in the CNS.

*Bax *deficiency also prevents apoptosis after axonal injury from a mechanical trauma [7,8] or ocular hypertension (glaucoma) [8]. Unlike during developmental apoptosis, *Bbc3 *deficiency did not change the time course of RGC death after axonal injury. This is perhaps not surprising since *Bim *deficiency [43] completely inhibited cell death in the RGC layer for 4 days after axotomy in explant culture. It is unclear if BAX activation in RGCs is completely dependent on BIM because longer time points were not examined. In hippocampal neurons both BIM and BBC3 can trigger cell death in response to the same insult [17,44]. It will be interesting to determine if *Bim *deficiency provides long-term protection to RGCs after axonal injury. If it does not, it is possible that BBC3 ultimately is capable of killing RGCs after axonal injury.

## Conclusion

BBC3-dependent BAX activation is required for developmental cell death of many retinal cell types. Since BBC3 appears to be critical for the developmental cell death of a number of different types of neurons, understanding how it is regulated will help to identify the molecular mechanisms that control neuronal number in the mammalian CNS. Unlike in developmental apoptosis, axonal injury-induced death of RGCs, BAX activation did not require BBC3. Thus, *in vivo *and in an insult-dependent manner, different BH3-only proteins control BAX activation even in the same neuronal cell type.

## Methods

### Animals

Null alleles of *Bbc3*^*tm1Gpz *^[45] (a generous gift from Gerard Zambetti), *Bim^tm1.1Ast ^*(the Jackson Laboratory stock number 004524) and *Bid^tm1Sjk ^*[46], were separately backcrossed at least 10 times into C57BL/6J prior to our receiving them. All colonies were maintained by intercrossing. Mice were maintained in a 12-hour light dark cycle and were fed chow and water ad libitum. All experiments were conducted in accordance with the Association for Research in Vision and Ophthalmology's statement on the use of animals in ophthalmic research and were approved by the University of Rochester's University Committee on Animal Resources.

### Tissue Collection, Plastic Sections and Flat mounts

Plastic sections were obtained as previously described [8,47]. Briefly, eyes were fixed by immersion in 2.5% gluteraldehyde and 2% paraformaldehyde at 4°C for 24 hours. Then eyes were dehydrated and embedded in technovit, 2.5 μm sections were cut, and sections that included the optic nerve were stained with cresyl violet. Retinal flat mounts were performed as previously described [8,47]. For RGC layer counts 2 40× fields from each retinal quadrant approximately 1 40× field from the margin of the retina were used for each retina. All cells within the field were counted except endothelial cells, which have an obvious elongated, non-neuronal morphology. For the total count for each retina, all eight fields were added together. To determine brain weights mice were weighed, anesthetized, and transcardially perfused with 4% paraformaldehyde. The entire brain was then removed and weighed.

### Immunohistochemistry and Cell Counts

Eyes were fixed in 4% paraformaldehyde in PBS at room temperature. The anterior segment was removed and the resultant eye cup was either processed for cryosectioning or whole mount staining. For immunohistochemistry on retinal sections, cryosections were blocked by incubating in 10% horse serum in 0.3% Triton X-100 in PBS, pH 7.3, for one hour at room temperature. Primary antibodies diluted in 0.1% Triton X-100 in PBS (PBST) containing 5% horse serum were then applied overnight at 4°C. The following day the sections were washed and treated for two hours with Alexafluor-conjugated secondary antibodies (Invitrogen) diluted in PBST. Each section was also counterstained with DAPI. GCL neuronal nuclei (DAPI), retinal ganglion cells (anti-POU4F1, Santa-Cruz, 1:200), cholinergic amacrine cells (anti-CHAT; Millipore, 1:200), and bipolar cells (anti-VSX2 (CHX10), Exalpha Biologicals, 1:200) were counted in sections. Only central sections located at or within 500 um near the optic nerve head (ONH) were used for counting. Within a section, each marker was counted in a 440 μm length of retina on each side of the ONH. This method was repeated on four sections per retina. For an individual retina the score was the average of the eight counts. Due to the fewer number of horizontal cells in the retina, calbindin D (CALB1, Sigma Aldrich, 1:1000) positive cells were counted over entire sections. Counts were performed at equivalent retinal eccentricities near the optic nerve head on four sections per retina. Dying cells (activated caspase 3 positive; R&D Systems, 1:500) in the developing retina on postnatal day 4 were also counted over the entire retinal section, similarly to the adult horizontal cell counts. Dopaminergic amacrine cells (anti-TH, Millipore, 1:500) and activated caspase 3 positive cells in adult retinas were counted in retinal flat mounts, since there are so few of these cells in the retina. For flat mounts, retinas were dissected free of the eye cup and blocked in PBST+10% horse serum for 4 hours. Retinas were incubated in primary antibodies diluted in PBST+5% horse serum for three overnights at 4°C. Counts were performed on two 20× fields from each quadrant approximately 220 μm from the peripheral edge of the retina (one half of a 20× field). Adult retinas were all between 6 weeks and 12 weeks of age. Four or more adult retinas of each genotype were quantified.

### Optic nerve injury

Controlled optic nerve crush (CONC) was performed on adult mice as previously described [8]. The optic nerve was crushed approximately 3-6 mm behind the eye for 5 seconds using self closing forceps (Roboz RS-5027). Retinas were harvested at various time points after the procedure. RGC layer counts after CONC were performed on nissl stained retinas as described above. Unmanipulated contralateral eyes or contralateral eyes that had a sham surgery performed (no crush of the optic nerve) were used as control eyes.

### Statistical Analysis

At least 3 animals were assessed for each genotype for all experimental conditions. For cell experiments involving quantification of results the experimenter was blinded to genotype and/or experimental group. ANOVA was used to compare between all genotypes. Upon finding statistical significant, multiple comparison tests were performed using the Tukey-Kramer method. P < 0.05 was considered significant.

## Competing interests

The authors declare that they have no competing interests.

## Authors' contributions

JH and RL designed the study and wrote the manuscript. JH carried out major parts of the experiments. All authors read and approved the final manuscript.
